# Ibrutinib combination therapy for advanced gastrointestinal and genitourinary tumours: results from a phase 1b/2 study

**DOI:** 10.1186/s12885-023-11539-1

**Published:** 2023-11-03

**Authors:** Do-Youn Oh, Maria Alsina Maqueda, David I. Quinn, Peter J. O’Dwyer, Ian Chau, Sun Young Kim, Ignacio Duran, Daniel Castellano, Jordan Berlin, Begona Mellado, Stephen K. Williamson, Keun-Wook Lee, Francisca Marti, Paul Mathew, Muhammad Wasif Saif, Ding Wang, Elizabeth Chong, Jacqueline Hilger-Rolfe, James P. Dean, Hendrik-Tobias Arkenau

**Affiliations:** 1 Seoul National University Hospital, Cancer Research Institute, Seoul National University College of Medicine, Seoul National University Graduate School, Seoul, South Korea; 2 Vall d’Hebron Institute of Oncology (VHIO), Barcelona, Spain; 3 University of Southern California Norris Comprehensive Cancer Center, Los Angeles, CA USA; 4 University of Pennsylvania, Philadelphia, PA USA; 5 The Royal Marsden NHS Foundation Trust-Royal Marsden Hospital, London, UK; 6 Asan Medical Center, University of Ulsan College of Medicine, Seoul, South Korea; 7 Hospital Universitario Marques de Valdecilla, IDIVAL, Santander, Spain; 8 Hospital Universitario, 12 de Octubre, Madrid, Spain; 9 Vanderbilt-Ingram Cancer Center, Nashville, TN USA; 10 Medical Oncology Department, Hospital Clinic i Provincial de Barcelona, IDIBAPS, University of Barcelona, Barcelona, Spain; 11 University of Kansas Hospital Cancer Center, Kansas City, KS USA; 12 Seoul National University College of Medicine, Seoul National University Bundang Hospital, Seongnam, South Korea; 13 The Christie NHS Foundation Trust, Manchester, UK; 14 Tufts Medical Center, Boston, MA USA; 15 Orlando Health Cancer Institute, Orlando, FL USA; 16 Henry Ford Hospital, Detroit, MI USA; 17 Pharmacyclics LLC, an AbbVie Company, South San Francisco, CA USA; 18 Sarah Cannon Research Institute – United Kingdom (SCRI-UK) and University College London, Cancer Institute, London, UK

**Keywords:** Renal cell carcinoma, Gastric adenocarcinoma, Colorectal carcinoma, Ibrutinib, Everolimus, Docetaxel, Cetuximab

## Abstract

**Background:**

Ibrutinib, a first-in-class inhibitor of Bruton’s tyrosine kinase, is approved for the treatment of various B-cell malignancies and chronic graft-versus-host disease. Based on encouraging preclinical data, safety and efficacy of ibrutinib combined with companion drugs for advanced renal cell carcinoma (RCC), gastric/gastroesophageal junctional adenocarcinoma (GC), and colorectal adenocarcinoma (CRC) were evaluated.

**Methods:**

Ibrutinib 560 mg or 840 mg once daily was administered with standard doses of everolimus for RCC, docetaxel for GC, and cetuximab for CRC. Endpoints included determination of the recommended phase 2 dose (RP2D) of ibrutinib in phase 1b and efficacy (overall response rate [ORR] for GC and CRC; progression-free survival [PFS] for CRC) in phase 2.

**Results:**

A total of 39 (RCC), 46 (GC), and 50 (RCC) patients were enrolled and received the RP2D. Safety profiles were consistent with the individual agents used in the study. Confirmed ORRs were 3% (RCC), 21% (GC), and 19% (CRC). Median (90% CI) PFS was 5.6 (3.9–7.5) months in RCC, 4.0 (2.7–4.2) months in GC, and 5.4 (4.1–5.8) months in CRC.

**Conclusions:**

Clinically meaningful increases in efficacy were not observed compared to historical controls; however, the data may warrant further evaluation of ibrutinib combinations in other solid tumours.

**Trial registration:**

ClinicalTrials.gov, NCT02599324.

**Supplementary Information:**

The online version contains supplementary material available at 10.1186/s12885-023-11539-1.

## Background

Ibrutinib, a first-in-class, once-daily covalent inhibitor of Bruton’s tyrosine kinase (BTK), is approved for the treatment of various B-cell malignancies and chronic graft-versus-host disease following the failure of one or more lines of systemic therapy and remains under investigation in these settings and for other diseases [1]. By binding a cysteine residue (Cys-481) near the adenosine triphosphate binding pocket of BTK, ibrutinib inhibits BTK activity [2]. Cysteine residues at analogous binding pocket positions have been identified in several kinases in the human genome, including the epidermal growth factor receptor (EGFR), human epidermal growth factor receptor 2 (HER2/neu), human epidermal growth factor receptor 4 (HER4/ErbB4), interleukin-2-inducible T-cell kinase (ITK), and Janus kinase 3. Consistent with this, preclinical data demonstrate that ibrutinib inhibits EGFR, HER2, and human epidermal growth factor receptor 3 (HER3) in breast cancer cell lines [3], and both EGFR (L858R) and EGFR (T790M) in mutant EGFR-expressing lung cancer cell lines [4]. Additionally, clinically relevant data show that ibrutinib inhibits ITK under physiologic conditions, potentially. Driven by ITK inhibition, shifts in helper T cell polarisation to a more anti-tumour functional phenotype in patients treated with ibrutinib may also enhance antitumour immune response [5]. Further, the disruption of BTK signaling itself may confer potential benefit by modifying the microenvironment of solid tumours, thereby improving their sensitivity to other drugs. For example, in pancreatic adenocarcinoma, BTK has been shown to play a role in mast cell degranulation [6] and has been shown to inhibit in vitro generation of myeloid-derived suppressor cells with resultant attenuation of microenvironmental immunosuppression and B-cell and macrophage-mediated CD8^+^T-cell suppression [7]. In addition, strong direct ibrutinib inhibition of epithelial and endothelial tyrosine kinase/bone marrow X kinase (ETK/BMX) may also play a role in renal cell carcinoma (RCC). ETK is highly expressed in RCC cell lines compared to normal renal cells [8] and increased ETK expression is positively correlated with higher clinical stage, grade, and metastasis [8]. Therefore, combining ibrutinib with other mechanistically complementary agents may confer increased antitumour activity and improve outcomes in patients with advanced tumours and limited treatment options.

There remains an unmet need for patients with RCC, gastric adenocarcinoma (GC), and colorectal adenocarcinoma (CRC) who have progressed on previous vascular endothelial growth factor (VEGF)-targeted and mammalian target of rapamycin (mTOR) inhibitors [9]. In RCC, treatment options have been limited due to toxicity and/or limited additional efficacy of combination therapy, including tyrosine kinase inhibitors and everolimus [10, 11]. Ibrutinib plus everolimus may represent a novel treatment approach for these patients who have failed other approved options. For patients with GC, prognosis for patients with advanced disease is poor, with a median overall survival of 10‒12 months [12]. However, combining taxanes with targeted agents has demonstrated increased activity compared to single-agent taxane therapy, suggesting that combining ibrutinib with docetaxel may provide clinically beneficial activity [13, 14]. Likewise, in patients with heavily pretreated, non-resectable CRC, survival outcomes are poor, ranging from 6.4 to 7.1 months with later-line treatments employed [15, 16]. Outcomes may be improved by combining drugs that target EGFR through different mechanisms of action [17].

On this basis, we conducted a phase 1b/2 clinical study to explore the safety, tolerability, and preliminary activity of ibrutinib combination therapy in previously treated patients with advanced solid tumours, including RCC, GC, CRC, and urothelial carcinoma (UC) who had failed multiple lines of therapy. Here we report results from the RCC, GC, and CRC cohorts (UC cohorts to be reported separately).

## Materials and methods

### Study design and patients

This was an open-label phase 1b/2 multicenter study (ClinicalTrials.gov; NCT02599324) conducted between December 2015 and March 2020, to determine the recommended phase 2 dose (RP2D) of ibrutinib combined with everolimus in RCC, docetaxel in GC, and cetuximab in CRC for previously treated patients. The data cutoff date for this analysis was 19 April 2021.

The phase 1b study followed a 3 + 3 + 3 design in each cohort to evaluate dose-limiting toxicities (DLTs) and determine the RP2D. The DLT observation period was 21 days following the initiation of combination therapy at the start of Cycle 1. A DLT was defined as any grade 3 or higher non-hematologic or grade 4 hematologic adverse event (AE) possibly related to either ibrutinib and/or drug combination that occurred during the DLT-observation period. DLTs were assessed in the first three evaluable patients at each dose level by a safety review committee and expanded to 6 or 9 patients if 1 of 3 or 2 of 6 patients experienced a DLT, respectively. The subsequent phase 2 portion of the study utilised a Simon’s 2-stage design in the GC and CRC cohorts. All patients were ≥ 18 years old with adequate hematologic, hepatic, and renal function and an Eastern Cooperative Oncology Group (ECOG) performance status of 0 or 1 (patients with RCC or CRC with an ECOG performance score of 2 were potentially acceptable after a discussion with the medical monitor). Patients had advanced (locally recurrent and/or metastatic) disease with histologically confirmed clear cell RCC, gastric or gastro-esophageal junctional adenocarcinoma or K-Ras and N-Ras wildtype (EGFR-expressing) CRC, with one or more measurable lesions per Response Evaluation Criteria in Solid Tumors (RECIST) version 1.1 guidelines. Patients were assessed by investigator to be a suitable candidate for the treatment partner (everolimus, docetaxel, or cetuximab) and ibrutinib as per their tumour type. To be eligible for the RCC cohort, patients had received between one and four prior lines of therapy in the advanced setting, including a VEGF-tyrosine kinase inhibitor. For the GC cohort, patients had received between one and three prior lines of therapy in the advanced setting, including a fluoropyrimidine regimen. For the CRC cohort, patients had received at least two and no more than four prior regimens in the advanced setting, which must have included both an irinotecan and oxaliplatin-based regimen unless unable to tolerate irinotecan chemotherapy. Key exclusion criteria included prior anti-cancer therapy within 28 days of the first dose of study drug (including neoadjuvant or adjuvant therapy if disease progression occurred at ≥ 12 months of treatment completion) and prior treatment with everolimus or temsirolimus (RCC cohort), any taxane (GC cohort), or cetuximab or panitumumab (CRC cohort).

All patients provided written informed consent for participation in this study as approved by the Institutional Review Board/Research Ethics Board/Independent Ethics Committee before any study-specific screening procedures were performed.

### Study treatment

In phase 1b, the starting dose level for ibrutinib was 560 mg orally once daily. Ibrutinib 560 mg was combined with everolimus 10 mg orally once daily (RCC cohort), docetaxel 60‒75 mg/m^2^ (dose according to local institutional policy) intravenously (IV) every 3 weeks (Q3W) (GC cohort), or cetuximab 400 mg/m^2^ IV (initial dose), then 250 mg/m^2^ weekly (CRC cohort) in 21-day cycles. Ibrutinib and partner agents were administered until unacceptable toxicity or disease progression at the prescribed doses in each cohort. In dose level 1 (ibrutinib 560 mg), if ≤ 22% of evaluable patients experienced a DLT during the first treatment cycle, patients were enrolled in dose level 2, in which the dose of ibrutinib was increased to 840 mg once daily in combination with the companion drug. If ≥ 33% of evaluable patients experienced a DLT at the 560 mg or 840 mg dose of ibrutinib, the dose was de-escalated to 420 mg or 560 mg once daily, respectively. Dose adjustment guidelines are described in supplemental methods.

Following the determination of the RP2D by a Dose Level Review Committee (DLRC), additional patients were enrolled and treated in phase 2 at the RP2D to further evaluate the efficacy of the regimen for each specified tumour type as prespecified.

### Study objectives

The primary objectives of phase 1b were to determine the RP2D for ibrutinib in each cohort: in combination with everolimus in RCC, docetaxel in GC, and cetuximab in CRC. Secondary objectives in phase 1b of each cohort included evaluation of the preliminary safety and tolerability, overall response rate (ORR) per RECIST v1.1, disease control rate (DCR; defined as complete response (CR), partial response (PR), or stable disease (SD) ≥ 6 weeks), duration of response (DOR), and pharmacokinetics of the combination regimens.

The primary objectives of phase 2 were to assess progression-free survival (PFS) in the RCC cohort and ORR in the GC and CRC cohorts. Secondary objectives in phase 2 included PFS in GC and CRC cohorts, ORR in RCC cohort, and DCR, DOR, overall survival (OS), safety, and tolerability in each cohort.

### Assessments and analyses

Tumour response was assessed using computed tomography (CT) or magnetic resonance imaging (in the case of CT contraindication). Imaging was performed at baseline and every 6 weeks thereafter, per the investigator using RECIST v1.1 guidelines, including confirmation of complete and PRs at least 28 days after the criteria for response were first met [18]. SD and disease progression were not confirmed. AEs were graded based on Common Terminology Criteria for Adverse Events version 4.03 [19]. All AEs were documented from the time of first dose of study treatment until 30 days following the last dose for ibrutinib or companion drug, whichever occurred later.

Plasma samples were collected for all patients for pharmacokinetic (PK) determination of ibrutinib in all cohorts and docetaxel in the GC cohort.

### Statistical considerations and analysis populations

For phase 1b, data were summarised by dose level for each cohort separately. For Phase 2, efficacy and safety data were summarised by RP2D dose level of ibrutinib for each cohort. The safety population included all patients who received at least one dose of any study drug. The DLT-evaluable population was defined as patients from phase 1b who completed ≥ 21 days of treatment or discontinued treatment before 21 days due to a DLT event. Efficacy analyses were performed in the efficacy-evaluable population, defined as eligible patients (including DLT-inevaluable) who received at least one dose of ibrutinib at the RP2D in combination with at least one dose of the companion drug and had at least 1 post-baseline tumour assessment, regardless of treatment duration, or had died prior to the first adequate post-baseline assessment (RCC cohort) or had measurable disease and at least 1 post-baseline tumour assessment (GC and CRC cohorts). When analysing efficacy at the RP2D, the data from patients in phase 1 treated with the RP2D phase 2 were merged prior to analysis.

For the RCC cohort, a single interim analysis for PFS futility was conducted when the twenty-fifth patient of 55 total dosed at the RP2D level had completed 6 months of follow-up. The study was designed to detect a 75% increase in median PFS to 8.6 months for ibrutinib plus everolimus with a sample size of 55 efficacy-evaluable patients. Phase 2 of the GC and CRC cohorts followed a Simon’s 2-stage design for patients treated at the RP2D. For the GC cohort, 39 patients were to be enrolled in two stages; if ≥ 2 of 21 patients had a tumour response (PR or CR) in stage 1, whereupon an additional 18 patients were enrolled in stage 2. Treatment was deemed acceptable for further clinical development if ≥ 6 patients responded. The Simon’s 2-stage design provided 80% power to test the historical ORR of 7% against the target ORR of 20%. In the CRC cohort, 40 patients were to be enrolled in two stages; if ≥ 3 of 22 patients were responders in stage 1, then an additional 18 patients were enrolled in stage 2. The Simon’s 2-stage design provided 80% power to test the historical ORR of 10% against the target ORR of 25%.

## Results

### RCC Cohort

#### Phase 1b

A total of 10 patients were enrolled in the RCC cohort in phase 1b; patients received everolimus with either 560 mg ibrutinib (*n* = 3) or 840 mg ibrutinib (*n* = 7); median duration of ibrutinib exposure was 2.9 months and median everolimus exposure was 2.6 months. One patient who received 840 mg ibrutinib plus everolimus was DLT-inevaluable due to dose interruption because of a non-DLT AE. Of the nine DLT-evaluable patients in phase 1b, one at dose level 2 (840 mg ibrutinib plus 10 mg everolimus) experienced a DLT consisting of diarrhea and nausea (both grade 3) for 18 days; thus the determined RP2D was 840 mg ibrutinib plus 10 mg everolimus both orally and once daily. Best response achieved was SD in 70% (*n* = 7/10).

#### Phase 1b/2

A total of 39 patients were enrolled in the RCC cohort at the RP2D in phase 1b/2, with enrollment discontinued based on interim futility analysis of patients completing 6 months of follow-up. Fifteen patients (39%) had received ≥ 3 prior lines of therapy and all had at least one metastatic site of disease (Table 1).

**Table 1:** Baseline patient and disease characteristics

	**RCC Cohort** ***N* = 39**	**GC Cohort** ***N* = 46**	**CRC Cohort** ***N* = 50**
**Median age (range), years**	62 (40–81)	58 (35–77)	64 (32–81)
≥ 65 years, *n* (%)	16 (41)	11 (24)	25 (50)
**Male, *n* (%)**	31 (79)	34 (74)	29 (58)
**Race, *n* (%)**^**a**^			
White	32 (82)	32 (70)	18 (36)
Black or African American	0	1 (2)	1 (2)
Asian	7 (18)	12 (26)	31 (62)
**ECOG performance status, *n* (%)**			
0	12 (31)	11 (24)	9 (18)
1	27 (69)	35 (76)	41 (82)
**Time from initial diagnosis to start of treatment, median (range), months**	33 (8–151)	13 (3–121)	38 (12–121)
**Metastatic sites of disease, *n* (%)**			
0	0	1 (2)	0
1	7 (18)	11 (24)	7 (14)
2	14 (36)	19 (41)	16 (32)
> 2	18 (46)	15 (33)	27 (54)
**Sites of metastasis, *n* (%)**			
With metastases	39 (100)	45 (98)	50 (100)
Bone	12 (31)	6 (13)	6 (12)
Liver	9 (23)	25 (54)	30 (60)
Lung	25 (64)	12 (26)	38 (76)
Lymph node	20 (51)	26 (57)	31 (62)
Peritoneal	6 (15)	20 (43)	12 (24)
**Number of prior regimens, *n* (%)**			
1	11 (28)	34 (74)	0
2	13 (33)	9 (20)	19 (38)
3	12 (31)	3 (7)	20 (40)
4	3 (8)	0	11 (22)

*CRC* colorectal adenocarcinoma, *ECOG* Eastern Cooperative Oncology Group, *GC* gastric adenocarcinoma, *RCC* renal cell carcinoma

^a^One patient in the GC cohort declined to answer/race unknown

Median (range) time on study in phase 1b/2 was 22.5 (0.5–37.4) months. Median (range) duration of ibrutinib exposure at the RP2D was 2.8 (0.1–27.9) months. The median average daily dose of ibrutinib was 641.7 mg/day and the median relative dose intensity was 76%. The most frequent reasons for discontinuation of ibrutinib were progressive disease (PD; 56%) and AEs (31%). Similarly, median (range) duration of everolimus exposure at the RP2D was 3.1 (0.1‒27.9) months. The median average daily dose of everolimus was 7.7 mg/day and the median relative dose intensity was 77%; 56% of patients discontinued everolimus due to PD and 28% due to an AE (Table 2).

**Table 2:** Summary of patient disposition for RP2D populations^a^

	**RCC Cohort** **Ibrutinib 840 mg + Everolimus** ***N* = 39**	**GC Cohort** **Ibrutinib 560 mg + Docetaxel** ***N* = 46**	**CRC Cohort** **Ibrutinib 840 mg + Cetuximab** ***N* = 50**
**Treatment duration, ibrutinib, median (range), months**	2.8 (0.1–27.9)	2.5 (0.1–15.1)	3.2 (0.2–14.0)
**Treatment duration of partner drug, median (range), months**	3.1 (0.1–27.9)	2.1 (0.0–14.5)	3.0 (0.0–13.8)
**Ibrutinib treatment disposition, *n* (%)**			
Still on treatment	0	0	0
Discontinued treatment	39 (100)	46 (100)	50 (100)
**Primary reason for ibrutinib discontinuation, *n* (%)**			
Disease progression	22 (56)	29 (63)	35 (70)
Clinical deterioration	2 (5)	3 (7)	1 (2)
Adverse events	12 (31)	8 (17)	8 (16)
Death	0	0	1 (2)
Withdrawal of consent	2 (5)	4 (9)	4 (8)
Investigator decision	1 (3)	2 (4)	1 (2)
**Companion drug treatment disposition, *n* (%)**			
Still on treatment	0	0	0
Discontinued treatment	39 (100)	46 (100)	50 (100)
**Primary reason for discontinuation of companion drug, *n* (%)**			
Disease progression	22 (56)	26 (57)	32 (64)
Clinical deterioration	2 (5)	2 (4)	1 (2)
Adverse events	11 (28)	10 (22)	10 (20)
Death	0	0	1 (2)
Withdrawal of consent	3 (8)	4 (9)	4 (8)
Investigator decision	1 (3)	4 (9)	2 (4)

*CRC* colorectal adenocarcinoma, *ECOG* Eastern Cooperative Oncology Group, *GC* gastric adenocarcinoma, *RCC* renal cell carcinoma, *RP2D* Recommended phase 2 dose

^a^Data cutoff date: 19 April 2021

**Fig. 1:**
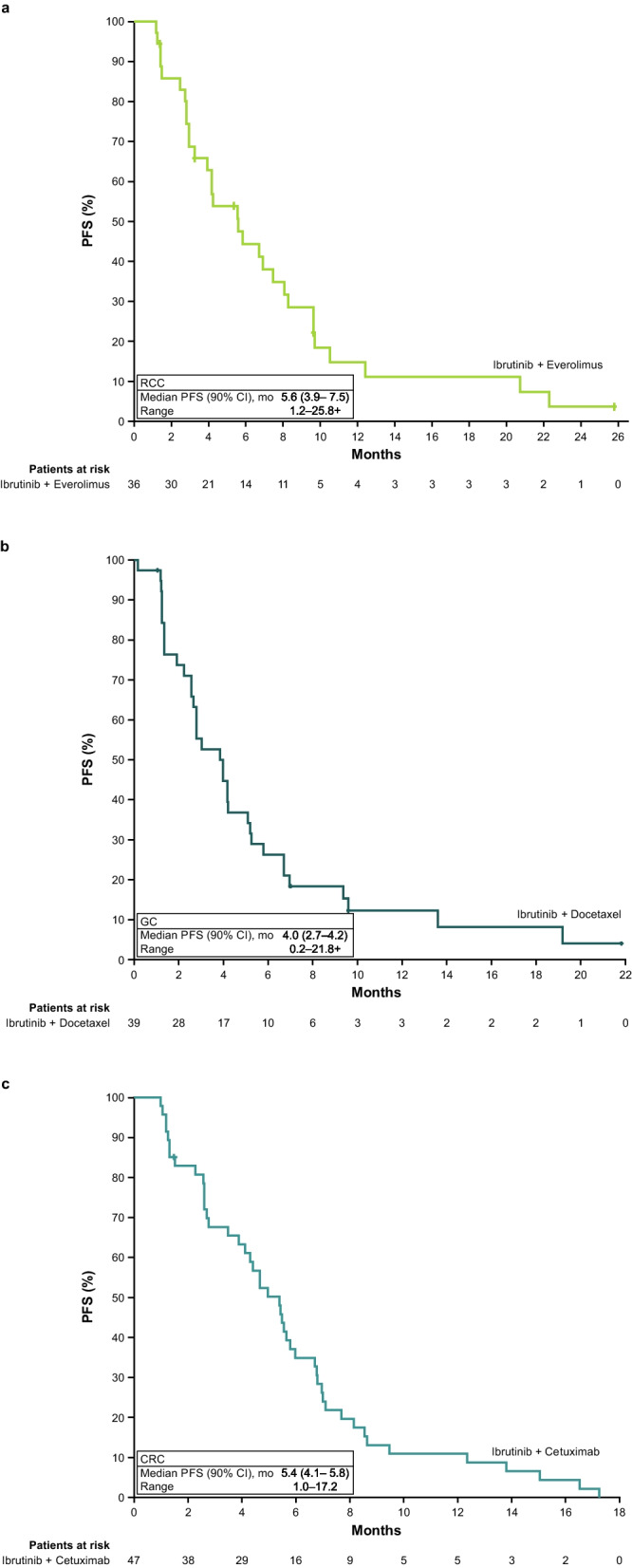
PFS per investigator assessment in the **a**) RCC cohort, **b**) GC cohort, and **c**) CRC cohort. CI, confidence interval; CRC, colorectal carcinoma; GC, gastric adenocarcinoma; PFS, progression-free survival; RCC, renal cell carcinoma

**Fig. 2:**
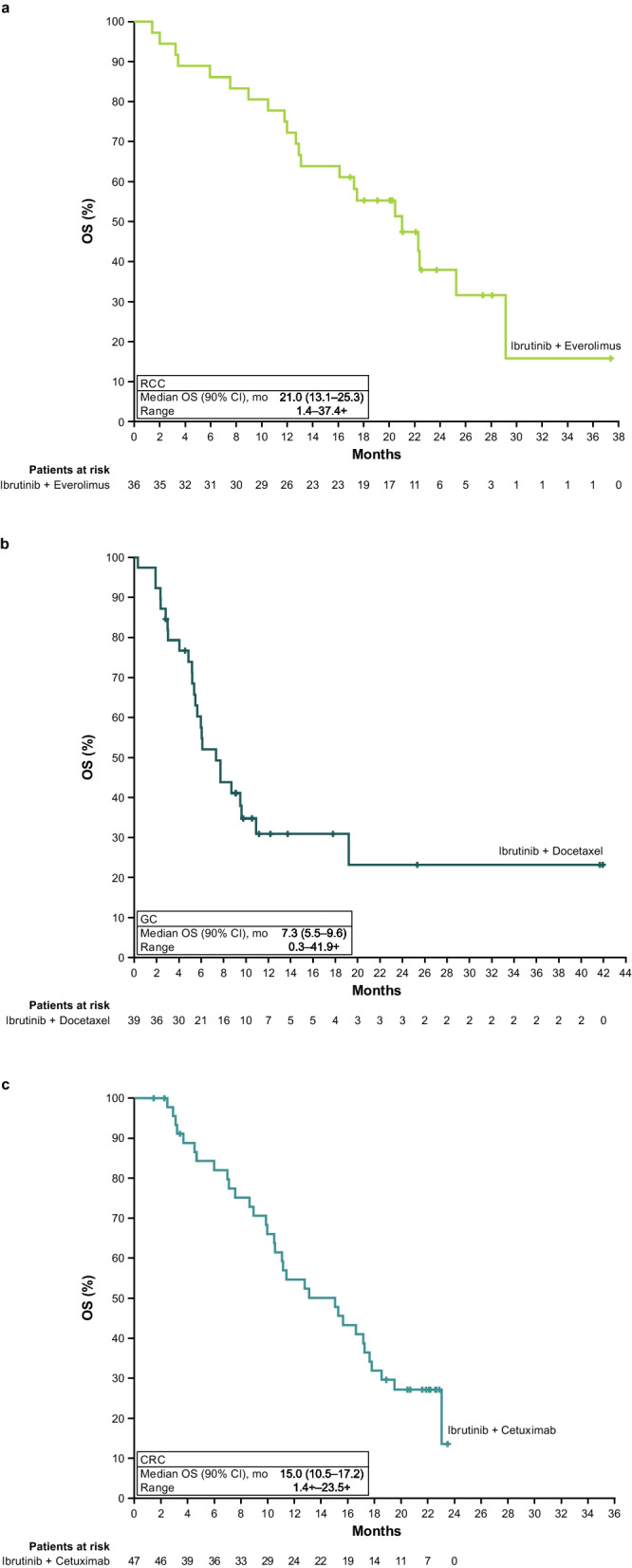
OS in the **a**) RCC cohort, **b**) GC cohort, and **c**) CRC cohort. CI, confidence interval; CRC, colorectal carcinoma; GC, gastric adenocarcinoma; OS, overall survival; RCC, renal cell carcinoma

Among efficacy-evaluable patients receiving the RP2D (*n* = 36 patients, 3 patients inevaluable with no post-baseline tumour assessment), median (90% CI) PFS was 5.6 (3.9–7.5) months. At 6 and 12 months, PFS rates were 44% and 15%, respectively (Fig. 1A). Confirmed ORR was 3%, with one patient achieving a confirmed PR. Best response was SD in 75% (*n* = 27/36) of patients and PD in 17% (*n* = 6/36) of patients. DCR (90% CI) was 81% (*n* = 29/36) (66.6%–90.5%). Response duration of the patient with PR was 3.1 months. Median (90% CI) OS was 21.0 (13.1–25.3) months (Fig. 2A).

The majority of patients treated at the RP2D experienced a TEAE (97%; *n* = 38/39), with grade ≥ 3 TEAEs occurring in 74% (*n* = 29/39) (Table 3). There were three deaths (*n* = 3/39; 8%) due to TEAEs (gunshot wound, hemoptysis, and RCC). The most common TEAEs of any grade included stomatitis (*n* = 25/39; 64%), diarrhea and epistaxis (*n* = 22/39; 56% each), and anemia (*n* = 19/39; 49%). The most common grade ≥ 3 events were anemia (*n* = 11/39; 28%), stomatitis (*n* = 7/39; 18%), hyperglycemia (*n* = 6/39; 15%), diarrhea (*n* = 5/39; 13%), and hypertension (*n* = 4/39; 10%) (Table 4).

**Table 3:** Safety summary

*n* (%)	RCC Cohort *N* = 39	GC Cohort *N* = 46	CRC Cohort *N* = 50
**TEAE (any grade)**	38 (97)	46 (100)	50 (100)
Grade ≥ 3 TEAE	29 (74)	42 (91)	34 (68)
**Patients with TEAEs leading to discontinuation of study treatment**^**a**^	13 (33)	15 (33)	11 (22)
Ibrutinib only	2 (5)	5 (11)	2 (4)
Companion drug only	1 (3)	5 (11)	2 (4)
Both ibrutinib and companion drug	10 (26)	5 (11)	7 (14)

^a^Includes adverse events with action taken as study treatment permanently withdrawn

*CRC* colorectal adenocarcinoma, *ECOG* Eastern Cooperative Oncology Group, *GC* gastric adenocarcinoma, *RCC* renal cell carcinoma, *TEAE* treatment-emergent adverse event

**Table 4:** TEAEs by cohort (any grade, ≥ 20% or grade ≥ 3, ≥ 10%)^a^

**RCC Cohort** ***N*** **= 39**		
**TEAE**	**Any Grade (≥ 20%)**	**Grade ≥ 3 (≥ 10%)**
Stomatitis	25 (64)	7 (18)
Diarrhea	22 (56)	5 (13)
Epistaxis	22 (56)	0
Anemia	19 (49)	10 (26)
Decreased appetite	17 (44)	0
Fatigue	16 (41)	1 (3)
Nausea	16 (41)	2 (5)
Asthenia	11 (28)	1 (3)
Cough	11 (28)	0
Thrombocytopenia	10 (26)	1 (3)
Vomiting	10 (26)	2 (5)
Edema peripheral	9 (23)	0
Dizziness	9 (23)	0
Rash maculo-papular	9 (23)	2 (5)
Blood creatinine increased	8 (21)	0
Hyperglycemia	8 (21)	6 (15)
Pyrexia	8 (21)	0
Hypertension	4 (10)	4 (10)
**GC Cohort** ***N* = 46**		
**TEAE**	**Any Grade (≥ 20%)**	**Grade ≥ 3 (≥ 10%)**
Anemia	27 (59)	16 (35)
Diarrhea	25 (54)	2 (4)
Neutropenia	20 (43)	20 (43)
Decreased appetite	19 (41)	2 (4)
Nausea	17 (37)	1 (2)
Stomatitis	17 (37)	1 (2)
Fatigue	16 (35)	1 (2)
Vomiting	15 (33)	0
Alopecia	14 (30)	0
Asthenia	14 (30)	5 (11)
Febrile neutropenia	14 (30)	14 (30)
Abdominal pain	10 (22)	3 (7)
Constipation	10 (22)	0
Neutrophil count decreased	10 (22)	9 (20)
White blood cell count decreased	9 (20)	5 (11)
Neutropenic sepsis	5 (11)	5 (11)
Pneumonia	6 (13)	5 (11)
**CRC Cohort** ***N* = 50**		
**TEAE**	**Any Grade (≥ 20%)**	**Grade ≥ 3 (≥ 10%)**
Dermatitis acneiform	38 (76)	12 (24)
Stomatitis	26 (52)	5 (10)
Dry skin	24 (48)	1 (2)
Diarrhea	23 (46)	3 (6)
Fatigue	20 (40)	2 (4)
Paronychia	19 (38)	1 (2)
Pruritus	16 (32)	1 (2)
Decreased appetite	14 (28)	1 (2)
Nausea	13 (26)	0
Palmar-plantar erythrodysaesthesia syndrome	13 (26)	3 (6)
Asthenia	11 (22)	3 (6)
Epistaxis	10 (20)	0

^a^Adverse events listed in descending order based on any grade incidence

*CRC* colorectal carcinoma, *GC* gastric adenocarcinoma, *RCC* renal cell carcinoma, *TEAE* treatment-emergent adverse event

### GC Cohort

#### Phase 1b

Twenty-one patients were enrolled in the GC cohort in phase 1b; all patients received 560 mg ibrutinib with docetaxel; median duration of ibrutinib exposure was 2.7 months and median docetaxel exposure was 1.4 months. Fourteen patients were DLT-inevaluable due to dose interruptions. Seven patients were DLT-evaluable. Two of these seven had DLTs (29%); one patient experienced grade 4 leukopenia for 3 days after receiving ibrutinib 560 mg QD plus docetaxel 75 mg/m^2^ Q3W; one patient had leukopenia for 7 days after receiving ibrutinib 560 mg QD plus 75 mg/m^2^ docetaxel Q3W. The recommended RP2D of ibrutinib was 560 mg once daily plus docetaxel 60 to 75 mg/m^2^ Q3W. Among the 18 response-evaluable patients, three (17%) had a confirmed PR, 11 (61%) had SD, and four (22%) had PD. The DCR (90% CI) was 78% (*n* = 14/18) (56.1%–92.0%).

#### Phase 1b/2

A total of 46 patients were enrolled in the GC cohort and were treated at the RP2D in phase 1b/2. Twelve patients (26%) had received at least two prior lines of therapy and 15 (33%) had > 2 metastatic sites of disease (Table 1). Median (range) time on study in phase 1b/2 was 12.2 (0.3–41.9) months. Median (range) duration of ibrutinib exposure at the RP2D was 2.5 (0.1–15.1) months. The median average daily dose of ibrutinib was 499.2 mg/day and the median relative dose intensity was 89%. The most frequent reasons for discontinuation of ibrutinib were PD (63%) and TEAEs (17%). The median relative dose intensity of docetaxel was 91%. Forty-three patients received starting doses of 75 mg/m^2^ docetaxel and three received starting doses between 60–75 mg/m^2^. Twenty-two patients (48%) had docetaxel dose reductions due to an AE during the study; 57% of patients discontinued docetaxel due to PD and 22% due to AEs (Table 2).

Among patients who received the RP2D in the efficacy-evaluable population (*n* = 39, 7 inevaluable with no post-baseline tumour assessment), median (90% CI) PFS was 4.0 (2.7–4.2) months. At 6 and 12 months, the PFS (90% CI) rates were 26% (15.5%–38.5%) and 12% (5.1%–22.8%), respectively (Fig. 1B). Confirmed ORR (90% CI) was 21% (10.6%–34.0%), with eight patients achieving PR. SD was reported in 54% (*n* = 21/39), amounting to a DCR (90% CI) of 74% (*n* = 29/39) (60.4%–85.4%). PD was best response in 26% (*n* = 10/39) of patients. Median (90% CI) DOR was 5.5 (3.0–10.8) months and median (90% CI) OS 7.3 (5.5–9.6) months, respectively (Fig. 2B).

All 46 patients in the phase 1b/2 GC cohort treated at the RP2D experienced a TEAE, with grade ≥ 3 TEAEs occurring in 91% (*n* = 42/46) (Table 3). There were three deaths (*n* = 3/46; 7%) due to TEAEs: adenocarcinoma gastric, hematuria, and intestinal obstruction. The most common TEAEs of any grade included anemia (*n* = 27/46; 59%), diarrhea (*n* = 25/46; 54%), and neutropenia (*n* = 20/46; 43%). The most common grade ≥ 3 events were neutropenia (*n* = 20/46; 43%), anemia (*n* = 16/46; 35%), febrile neutropenia (*n* = 14/46; 30%), neutrophil count decreased (*n* = 9/46; 20%), neutropenic sepsis, pneumonia, and white blood cell count decreased (*n* = 5/46; 11% each) (Table 4).

### CRC Cohort

#### Phase 1b

Twenty patients were enrolled in the CRC cohort in phase 1b; 8 and 12 patients, respectively, received 560 mg and 840 mg ibrutinib with cetuximab. Median duration of ibrutinib exposure was 2.7 months and median cetuximab exposure was 2.3 months. Eleven patients were DLT-inevaluable, 10 due to dose interruption because of non-DLT AEs and one due to dosing non-compliance. Among the nine DLT-evaluable patients in this cohort, there were no reported DLTs. Therefore, the RP2D used in phase 2 was 840 mg ibrutinib orally daily plus 400 mg/m^2^ IV cetuximab, followed by 250 mg/m^2^ in subsequent weeks. Among the 18 response-evaluable patients, two patients (11%) achieved a PR and 12 patients (67%) had SD amounting to a DCR (90% CI) of 78% (*n* = 14/18) (56.1%, 92.0%).

#### Phase 1b/2

A total of 50 patients were enrolled in the CRC cohort in phase 1b/2. All patients had received at least two prior lines of therapy and 27 (54%) had > 2 metastatic sites of disease (Table 1). Median (range) time on study in phase 1b/2 was 22.1 (0.3–23.5) months. Median (range) duration of ibrutinib exposure at the RP2D was 3.2 (0.2–14.0) months. The median average daily dose of ibrutinib was 791.3 mg/day and the median relative dose intensity was 94.2%. The most frequent reason for discontinuation of ibrutinib was PD (70%); 16% discontinued due to AEs (Table 2). The median relative dose intensity of cetuximab was 90%; 64% of patients discontinued cetuximab due to PD and 20% due to AEs (Table 2).

Among patients who received the RP2D in the efficacy-evaluable population (*n* = 47, 3 were inevaluable with no post-baseline tumour assessment), median (90% CI) PFS was 5.4 (4.1–5.8) months. At 6 and 12 months, the PFS (90% CI) rates were 35% (23.7%–46.4%) and 11% (4.8%–19.8%), respectively (Fig. 1C). The confirmed ORR in the CRC cohort was 19%, with nine patients achieving a PR and SD in 30, amounting to a DCR (90% CI) of 83% (*n* = 39/47) (71.4%–91.2%). Median (90% CI) DOR was 11.1 (4.2–12.5) months and median (90% CI) OS was 15.0 (10.5–17.2) months (Fig. 2C). Median OS was 15.3 months for patients with left-sided tumours (*n* = 35) and 10.6 months for those with right-sided tumours (*n* = 14).

All 50 patients treated at the RP2D experienced a TEAE of any grade, with grade ≥ 3 TEAEs occurring in 68% (*n* = 34/50) (Table 3). One death due to pulmonary embolism occurred during phase 1b, outside of the DLT evaluable population, and was deemed unrelated to treatment.

The most common TEAEs of any grade included dermatitis acneiform (*n* = 38/50; 76%), stomatitis (*n* = 26/50; 52%), dry skin (*n* = 24/50; 48%), and diarrhea (*n* = 23/50; 46%). Common grade ≥ 3 TEAEs included dermatitis acneiform (*n* = 12/50; 24%) and stomatitis (*n* = 5/50; 10%) (Table 4).

### Pharmacokinetics

Ibrutinib was rapidly absorbed with a median time to maximum concentration (t_max_) of 2.03 to 3.89 h and mean apparent terminal half-life (t_1/2_) ranged from 5 to 6 h. Mean ibrutinib steady-state C_max_ and AUC_0-24 h_ for RCC and CRC cohorts were 351 ng/mL and 2568 ng∙h/mL and 371 ng/mL and 2807 ng∙h/mL, respectively, at the RP2D dose of 840 mg ibrutinib in combination with everolimus or cetuximab. Ibrutinib exposures at the RP2D dose of 840 mg in patients with RCC and CRC were comparable regardless of co-medications (everolimus or cetuximab). Mean ibrutinib steady-state C_max_ and AUC_0-24 h_ for GC cohort were 164 ng/mL and 1253 ng∙h/mL, respectively at the RP2D of 560 mg. In the GC cohort, docetaxel C_max_ ranged from 467 to 38,000 ng/mL following administration of 53.9 to 75.8 mg/m^2^ docetaxel with ibrutinib at the RP2D of 560 mg. The mean concentration (% coefficient of variation) of dose normalised docetaxel C_max_ following exclusion of the high outlier concentration observed in one patient was 34.6 ng/mL (60%).

## Discussion

This multicenter, open-label, phase 1b/2 study of ibrutinib combination therapy in patients with RCC (ibrutinib plus everolimus), GC (ibrutinib plus docetaxel), or CRC (ibrutinib plus cetuximab) demonstrated acceptable safety in patients with advanced tumours. Overall, results presented here suggest that administration of ibrutinib 840 mg orally with everolimus 10 mg in RCC, ibrutinib 560 mg with docetaxel 60 to 75 mg/m^2^ Q3W in GC, and ibrutinib 840 mg with cetuximab 400 mg/m^2^, then 250 mg/m^2^ weekly in CRC, each administered in a similar (or later line) than as recommended by consensus guidelines at the time of study initiation, were feasible but did not result in a meaningful increase in efficacy when compared to historical controls in each disease setting.

In metastatic RCC, other combinations, such as sorafenib plus everolimus, have been tested. Nevertheless, excessive gastrointestinal and skin toxicity limited the translation of this combination to the clinical trials. As single agents, everolimus and sorafenib reported PFS of 4.9 and 5.5 months [20–22], respectively. In the current study, the combination of ibrutinib plus everolimus demonstrated a similar median PFS (5.6 months), which does not suggest an additive or synergistic effect. However, the patient populations are different, making comparisons across studies difficult.

In both the GC and CRC cohorts, 14 and 11 patients were DLT-inevaluable, suggesting a sick population overall at study entry. Nonetheless, in patients with refractory GC, ibrutinib plus docetaxel was associated with a higher ORR of (18%) relative to single-agent docetaxel (7%) but a similar 6-month PFS (29% and 26%) [23] and lower ORR and PFS relative to results in second-line patients with paclitaxel plus ramucirumab (ORR of 28% and 6-month PFS of 36%) [14]. A previous study in patients with refractory CRC treated with cetuximab as third-line therapy reported a similar response rate (12%) compared with the current study (15%) but with a shorter median PFS (1.4 and 5.4 months, respectively) and OS (6.6 and 15.0 months, respectively) [24]. Other studies in patients with advanced CRC reported median OS of 6.4 and 7.1 months with regorafenib monotherapy and TAS-102, respectively [15, 16]. In the current study, median OS among patients with left-sided versus right-sided tumours (15.3 vs 10.6 months) aligns with previous reports of longer survival in those with left-sided tumours treated with anti-EGFR therapies [25–27].

The safety profiles for each of the drug combinations were generally consistent with the known safety profiles for the individual agents used in the study, suggesting no significant interaction between ibrutinib and partner agents and were as expected for patients with these advanced tumour types considering prior therapies and treatment duration. TEAEs related to companion drugs in this study were consistent with those reported previously in everolimus in RCC, [28] docetaxel in GC, [29] and cetuximab in CRC, [30] respectively. Notably, 14 patients (30%) in the GC cohort experienced grade ≥ 3 febrile neutropenia, which is a higher incidence than might have been expected. The explanation for this finding is not immediately apparent, but interpretation is potentially confounded by the heavily pre-treated nature of this patient population, the problematic toxicity of docetaxel in this clinical setting, and the relatively small number of treated patients. No pharmacokinetic interactions were identified with these combination treatment regimens for ibrutinib (all cohorts) or docetaxel (GC cohort) levels.

The heavily pretreated patient populations in this study may not have been the optimal contexts for evaluation of novel ibrutinib combination regimens, as evidenced by the modest efficacy observed relative to historical controls. However, the generally manageable safety profile seen across all three cohorts and the high levels of relative dose delivery of both ibrutinib and partner agents support the feasibility of the regimens studied, thereby warranting consideration of further evaluation in earlier stage settings and/or with different partner agents.

### Supplementary Information

**Additional file 1: Supplementary Methods.** Follow-up. **Supplementary Methods.** Maximum tolerated dose. **Supplementary Methods.** Dose adjustment guidelines. **Supplementary Table S1.** Prior therapies

## Data Availability

Requests for access to individual participant data from clinical studies conducted by Pharmacyclics LLC, an AbbVie Company, can be submitted through Yale Open Data Access (YODA) Project site at http://yoda.yale.edu. Requests for the datasets used and/or analysed during the current study can be made directly with the study sponsor via the following link: https://vivli.org/ourmember/abbvie/.

## Abbreviations

AE: Adverse event
AUC: Area under the curve
BTK: Bruton’s tyrosine kinase
CR: Complete response
CRC: Colorectal adenocarcinoma
CT: Computed tomography
DCR: Disease control rate
DLRC: Dose Level Review Committee
DLT: Dose-limiting toxicity
DOR: Duration of response
ECOG: Eastern Cooperative Oncology Group
EGFR: Epidermal growth factor receptor
ETK/BMX: Endothelial tyrosine kinase/bone marrow X kinase
GC: Gastric/gastroesophageal junctional adenocarcinoma
HER2/neu: Human epidermal growth factor receptor 2
HER3: Human epidermal growth factor receptor 3
HER4/ErbB4: Human epidermal growth factor receptor 4
ITK: Interleukin-2-inducible T-cell kinase
IV: Intravenously
mTOR: Mammalian target of rapamycin
ORR: Overall response rate
OS: Overall survival
PD: Progressive disease
PR: Partial response
PFS: Progression-free survival
QD: Daily
Q3W: Every 3 weeks
RCC: Renal cell carcinoma
RECIST: Response Evaluation Criteria in Solid Tumors
RP2D: Recommended phase 2 dose
SD: Stable disease
TEAE: Treatment-emergent adverse event
UC: Urothelial carcinoma
VEGF: Vascular endothelial growth factor
