# Supplementary material for: Ibrutinib combination therapy for advanced gastrointestinal and genitourinary tumours: results from a phase 1b/2 study
Source: BMC Cancer. 2023 Nov 3;23:1056. doi: 10.1186/s12885-023-11539-1 (PMC10623721; doi:10.1186/s12885-023-11539-1)
Supplement: Supplementary file 1 — Additional file 1: Supplementary Methods. Follow-up. Supplementary Methods. Maximum tolerated dose. Supplementary Methods. Dose adjustment guidelines. Supplementary Table S1. Prior therapies [file 12885_2023_11539_MOESM1_ESM.docx]

**Supplementary Information**

**Ibrutinib Combination Therapy for Advanced Gastrointestinal and Genitourinary Tumours: Results From a Phase 1b/2 Study**

Do-Youn Oh, MD, PhD, Maria Alsina Maqueda, MD, PhD, David I. Quinn, MD, PhD, Peter J. O’Dwyer, MD, Ian Chau, MD, Sun Young Kim, MD, PhD, Ignacio Duran, MD, PhD, Daniel Castellano, MD, Jordan Berlin, MD, Begona Mellado, MD, PhD, Stephen K. Williamson, MD, Keun-Wook Lee, MD, PhD, Francisca Marti, MD, PhD, Paul Mathew, MD, Muhammad Wasif Saif, MD, Ding Wang, MD, PhD, Elizabeth Chong, PhD, Jacqueline Hilger-Rolfe, PhD, James P. Dean, MD, PhD, Hendrik-Tobias Arkenau, MD, PhD

**Table of Contents**

**Supplementary Methods.** Follow-up 2

**Supplementary Methods.** Maximum tolerated dose 2

**Supplementary Methods.** Dose adjustment guidelines 2

**Supplementary Table S1.** Prior therapies. 3

**Supplementary Methods**

Patients were followed every 3 months after study treatment ended.

Maximum tolerated dose

In a previous phase 1 study, in which patients received up to 12.5 mg/kg/day (1400 mg/day) ibrutinib, no maximum tolerated dose was reached (Advani RH et al. *J Clin Oncol.* 31:1:88‒94).

Dose adjustment guidelines

Dose modifications were not permitted in phase 1b in the absence of a DLT. However, the dose of ibrutinib was modified in cases of the following: grade 4 neutropenia (ANC <500/μL) for >7 days; grade 3 thrombocytopenia (platelets <50,000/μL) with clinically significant bleeding events; grade 4 thrombocytopenia (platelets <25,000/μL); grade 3 or 4 nausea, vomiting, or diarrhea if persistent despite optimal anti-emetic and/or anti-diarrheal therapy; any other grade 4 or unmanageable grade 3 toxicity. For everolimus, a 50% reduction in the previously administered daily dose was recommended for the management of severe or intolerable adverse reactions. For further details of dosage reductions for everolimus related toxicity, reference should be made to the everolimus prescribing information (AFINITOR [everolimus] tablets for oral use [package insert]. East Hanover, NJ: Novartis Pharmaceuticals Corporation; 2021). For docetaxel, a reduction from 75 mg/m^2^ to 60 mg/m^2^ was recommended in cases of neutropenia or grade 4 thrombocytopenia. For cetuximab, dose was delayed at the first occurrence of severe acneiform rash, reduced from 250 mg/m^2^ to 200 mg/m^2^ at the second occurrence, and further reduced to 150 mg/m^2^ at the third occurrence.

**Table S1. Prior therapies**

| **RCC Cohort** | ***N* = 39** |
| --- | --- |
| **Prior agent(s) in metastatic setting, *n* (%)** |  |
| VEGF-targeting TKI^a^ | 34 (87) |
| Checkpoint inhibitor | 21 (54) |
| Other TKIs^b^ | 6 (15) |
| Others^c^ | 2 (5) |
| Other anti-angiogenics^d^ | 3 (8) |
| Cytokines^e^ | 1 (3) |
| **Prior radiation, *n* (%)** |  |
| Yes | 7 (18) |
| No | 32 (82) |
| **GC Cohort** | ***N* = 46** |
| **Prior regimen(s) in advanced setting, *n* (%)** |  |
| Platinum agents | 38 (83) |
| Fluoropyrimidines | 37 (80) |
| Anti-HER2 agents (trastuzumab) | 11 (24) |
| Folinic acid | 11 (24) |
| Anthracyclines | 9 (20) |
| Others^c^ | 6 (13) |
| Checkpoint inhibitors | 5 (11) |
| FOLFOX | 4 (9) |
| Anti-angiogenics (ramucirumab, apatinib) | 2 (4) |
| Topoisomerase inhibitors (irinotecan) | 2 (4) |
| **Prior radiation, *n* (%)** |  |
| Yes | 6 (13) |
| No | 40 (87) |
| **Prior surgery, *n* (%)** |  |
| Yes | 32 (70) |
| No | 14 (30) |
| **CRC Cohort** | ***N* = 50** |
| **Prior regimen(s) in metastatic setting, *n* (%)** |  |
| Irinotecan | 49 (98) |
| Fluoropyrimidines | 48 (96) |
| Oxaliplatin | 43 (86) |
| Folinic acid | 43 (86) |
| Anti-angiogenics (bevacizumab, ramucirumab, afilbercept) | 38 (76) |
| Other cytotoxics (gemcitabine, mitomycin, pemetrexed) | 9 (18) |
| Others^c^ | 4 (8) |
| Checkpoint inhibitors | 2 (4) |
| Anti-EGFR agents | 2 (4) |
| XELOX, XELIRI | 2 (4) |
| Multi-TKIs (regorafenib) | 1 (2) |
| **Prior radiation, *n* (%)** |  |
| Yes | 20 (40) |
| No | 30 (60) |
| **Prior surgery, *n* (%)** |  |
| Yes | 47 (94) |
| No | 3 (6) |

CRC, colorectal adenocarcinoma; EGFR; epidermal growth factor receptor; FOLFOX, folinic acid, fluorouracil, oxaloplatin; GC, gastric adenocarcinoma; RCC, renal cell carcinoma; TKI, tyrosine kinase inhibitor.

^a^Includes sorafenib, sunitinib, axitinib, pazopanib, or tivozanib.

^b^Includes crizotinib and cabozantinib.

^c^Refers to investigational agents/clinical trials.

^d^Includes bevacizumab.

^e^Includes interleukin-2 and interferon-alfa.
